# Synbiotic Supplementation Improves Metabolic Factors and Obesity Values in Women with Polycystic Ovary Syndrome Independent of Affecting Apelin Levels: A Randomized Double-Blind Placebo - Controlled Clinical Trial 

**DOI:** 10.22074/IJFS.2021.6186

**Published:** 2021-01-27

**Authors:** Sima Darvishi, Maryam Rafraf, Mohammad Asghari-Jafarabadi, Laya Farzadi

**Affiliations:** 1 Student’s Research Committee, Department of Community Nutrition, Faculty of Nutrition and Food Science, Tabriz University of Medical Sciences, Tabriz, Iran; 2 Nutrition Research Center, Department of Community Nutrition, Faculty of Nutrition and Food Science, Tabriz University of Medical Sciences, Tabriz, Iran; 3 Road Traffic Injury Research Center, Tabriz University of Medical Sciences, Tabriz, Iran; 4 Department of Statistics and Epidemiology, Faculty of Health, Tabriz University of Medical Sciences, Tabriz, Iran; 5 Department of Obstetrics and Gynecology, Faculty of Medicine, Tabriz University of Medical Sciences, Tabriz, Iran

**Keywords:** Apelin, Metabolic Factors, Obesity, Polycystic Ovary Syndrome, Synbiotic

## Abstract

**Background:**

This research investigated the symbiotic supplement influences on serum glycemic indices and lipids as well as apelin rates and obesity values in polycystic ovary syndrome (PCOS) patients.

**Materials and Methods:**

A total of 68 obese or overweight patients (20-44 years old) with PCOS were enrolled to conduct a randomized double-blinded placebo-controlled clinical trial. A total of 34 people in the synbiotic group re- ceived a synbiotic supplement and 34 people in the placebo group received placebo, daily for 8 weeks. Fasting blood specimens, anthropometric measurements and dietary intake data were gathered three times during the study. The information was analyzed by independent t test, paired t test, analysis of covariance and chi-square test.

**Results:**

Synbiotic supplementation significantly decreased serum fasting glucose (P=0.02), insulin (P=0.001), ho- meostatic model assessment for insulin resistance (IR, P=0.001), weight (P=0.02), body mass index (BMI, P=0.02), waist circumference (WC, P=0.01), hip circumference (HC, P=0.02), and waist-to-height ratio (WHtR, P=0.02) but significantly increased high-density lipoprotein (HDL) cholesterol (P=0.02) compared to the placebo. At the end of the trial, no significant differences were seen in serum total cholesterol, triglyceride (TG), low-density lipoprotein (LDL) cholesterol, or apelin levels as well as waist-to-hip ratio (WHR) between the two groups.

**Conclusion:**

Synbiotic supplementation improved glycemic indices, lipid profile and obesity values in women with PCOS. These beneficial effects were not related with alterations in serum apelin levels (Registration number: IRCT20100408003664N19).

## Introduction

Polycystic ovary syndrome (PCOS) is a momentous endocrine disarray in reproductive age women that leads to infertility and an enhancement in the occurrence of abortion, gestational diabetes and pre-eclampsia (1, 2). The prevalence of PCOS is estimated to be 4 to 21% worldwide (3). These patients indicate an irregular menstruation period, an ovulatory cycle, and androgen excess (4).

PCOS is contemplated as a multifactorial disease that is often accompanied by metabolic disorders including obesity, insulin resistance (IR), dyslipidemia and increased levels of androgens. PCOS is a risk factor for type 2 diabetes, cardiovascular difficulties and endometrial cancers (2, 5).

More than 50% of patients with PCOS are obese (6). Adipose tissue generates several proteins that are called adipokines that have a hormonal function (7). Studies have shown that adipokines derived from fatty tissue, can contribute to the pathogenesis of PCOS (8). Apelin (APLN-13or-17) is an adipokine located on the Xq25- q26 chromosome and it contains 77 amino acids. Adipose tissue is not the only determinant of serum apelin levels. Other organs such as the ovary, breast, gastrointestinal system, and central nervous system can also contribute to apelin secretion (9). It has been proposed that apelin has a function in regulating glucose metabolism, lipolysis and food intake (10). Some studies stated that apelin has a significant function in controlling IR in diabetes mellitus type 2 mice and humans (11, 12).

The implication of apelin in the pathogenesis of PCOS seems to be more intricate, involving a disrupted synthesis of androgens (13, 14). Lifestyle modification such as weight loss and using oral contraceptive pills and metformin are the most common treatments for PCOS (5, 15). Recently, modulation of intestinal microbiota equilibrium using probiotics and prebiotics has been suggested as an effective approach for some diseases such as PCOS. The intestinal microbiota imbalance can lead to increased ovarian androgen production and prevents the spread of the natural follicles of the ovary through chronic inflammatory response and IR (15). Living microorganisms called probiotics concede a well profit up on the hostess until used with adequate quantities (16). On the other hand, prebiotic is as "a non-digestible food component" that lucratively affects the host via selectively promoting the development and/or activity of one or a confined number of bacteria in the colon (17). 

A composition of probiotic and prebiotic that augment the viability of probiotics in the intestinal tract by stimulating growth or betterment metabolic activity, is called symbiotic (18). Samimi et al. (19) presented that synbiotic supplementation for 12 weeks, declined IR and serum levels of insulin, very-low-density lipoprotein (VLDL) cholesterol and triglyceride (TG) in PCOS patients. Karimi et al. (20) reported that synbiotic supplementation at a dose of 1000 mg for 12 weeks, reduced serum apelin-36 levels in PCOS patients, although changes in IR, blood glucose and insulin levels were not significant. In another study, synbiotic supplementation for 12 weeks led to a notable reduction in serum insulin levels and IR in patients with PCOS (21). There is no further study about the effect of synbiotics in patients with PCOS.

Since variations in gut microbiota combination have been reported in subjects with PCOS (22), synbiotics can be considered as remedial agents (15). However, controversies have been seen in the outcomes of studies (19, 21). Moreover, the effects of synbiotics supplementation on obesity values have not been assayed in these patients. Accordingly, we designed a study to assess the effects of synbiotics on glycemic indices, serum lipids, and apelin levels and anthropometric values in PCOS patients.

## Materials and Methods

In this randomized double-blinded placebo-controlled clinical trial study, out of 68 people suffering from PCOS within the age range 20-44 years and with body mass index (BMI) ranged 25-40 kg/m^2^ cooperated in this randomized double-blinded placebo-controlled clinical trial in Alzahra Hospital in General Gynaecology Clinic Department, in Tabriz, between June and December 2018. 

The sample size was calculated based on information obtained from the research conducted by Ahmadi et al. (23) on IR. The sample size was calculatedas 30 in each group, for the confidence intervals of 95% and a power of 80%. The projected dropout rate was set at 34 with the increase in sample size per group. 2003 Rotterdam criteria determined cognitive performance in PCOS due to three dimensions such as: idiopathic amenorrhea oroligomenorrhea, presenting the hyperandrogenism (convenient clinical and/or biochemical assessments) and PCOS via sonography (8).

Criteria for exclusion included: thyroid gland disorders, diabetes, high levels of serum prolactin (hyperprolactinemia), gestation and lactation, liver or kidney disease, Cushing’s syndrome diseases, cardiovascular diseases, high blood pressure, drug consumption including hydrochlorothiazide, insulin therapy, using beta blockers, low-density lipoprotein (LDL) cholesterol medicine, addiction, fertility treatments available, using cortisone-like medicine, following a special diet as well as being athlete or sport in orderly array (longer than the standard 2-week), antibiotic use during the last month and at the time of the study, use of any dietary supplements in the last 2 months or throughout the study, receiving probiotics, prebiotics and synbiotics in the last three months and simultaneous of the study, regular consumption of probiotic products and sensitivity to symbiotic orprobiotic capsules.

The research protocol was approved by the Research Ethics Committee of Tabriz University of Medical Sciences (code: IR.TBZMED.REC.1396.1080) and registered at the IRCT website (Registration number: IRCT20100408003664N19). Written informed consent was gained from all participating women before the study.

Based on the age and BMI, the participants were randomly divided into two groups by a size 2 block randomization technique. In this technique, patients had to retain a normal diet and physical activity throughout the trial.

Public information was obtained for each participant. Body weight and height were measured using a scale (Seca, Germany), and a mounted tape, respectively. BMI was estimated by dividing the weight in kilogram by height in (m)^2^ . Soft measuring tape in standing up position was used to obtain hip circumference (HC) as well as waist circumference (WC) (24). WC was measured in the narrowest section across the costaarch and the crest of the iliumand HC as the distance around the largest part of hips. Waist-to-hip ratio (WHR) and waist-to-height ratio (WHtR) were respectively calculated as follows: WC in centimeter divided by HC in centimeter and WC in centimeter divided by height in centimeter.

Validated International Assessment of Physical Activity Questionnaire (IPAQ) was used to estimate the level of physical activity (25). 

Also, 24-hour method was used to acquire data on daily intake of energy and macronutrients (2 week days and 1 weekend day). Questionnaires were completed before starting the study, at the end of the fourth week and the end of the study. The average energy and macronutrients intakes of all patients were analyzed using Nutritionist 4 software (FDBInc., California).

For eight weeks, patients in the treatment group (n=34) received one capsule of synbiotic and placebo group (n=34) received placebo capsule that was essential in daily use after lunch

Each synbiotic capsule (500 mg, Zist-Takhmir Co., Iran), included seven strains of helpful bacteria (Lactobacillus casei 3×10^9^ colony-forming units (CFU)/g, genus Lactobacillus *Lactobacillus rhamnosus* 7×10^9^ CFU/g, *Lactobacillus bulgaricus* 5×10^8^ CFU/g, genus Lactobacillus acidophilus 3×1010 CFU/g, *Bifidobacterium longum* subsp1×10^9^ CFU/g, (strainACS-071-V-Sch8b) 2×10^10^ CFU/g and *Streptococcus thermophilus* subsp3×10^8^ CFU/g and inulin-type prebiotics (Fructooligosaccharides (FOS)). The placebo capsule contained starch with identical color and form. 

The compliance of the participants with the study protocol was evaluated via phone talks once per week and by assessment of returned capsules every 2 weeks.

### Blood sampling and biochemical assays

Blood samples (5 mL) were collected after 12-hour overnight fasting, in the morning. The serum was separated via centrifugation and stored at -70°C up to subsequent research. The standardized enzymatic method using a commercially available Kit (Pars Azmoon, Iran) was employed to evaluate blood glucose. ELISA method using Monobind kit (Monobind Inc., CA, USA) was used to measure the serum insulin level and IR was defined via Homeostasis Model Assessment (HOMA) equations using the following relation: HOMA-IR was estimated by multiplying the fasting insulin (μIU/mL) and fasting glucose (mg/dL) divided by 405 (26).

Standardized enzymatic approach using a commercially available Kit (Pars Azmoon, Iran) was employed to evaluate the total blood cholesterol (TC), TG, and high-density lipoprotein (HDL) cholesterol. Serum concentration of LDL cholesterol was quantified via Friedewald formula (FF): LDL cholesterol=TC-(HDL cholesterol+TG/5) (27). Enzymelinked Immunosorbent Assay (shortened as ELISA) using Mediagnost kit (Cat No. E2037Hu; Shanghai Crystal DayBiotech Co., Ltd) was performed to specify blood apelin ratio. The inter-and intra-assay coefficients of variation toward apelin were considered lower than 8 and 10%, respectively.

Finally, all the body measurements, and biochemistry assessments were reassessed at the end of the study

### Statistical analysis

Statistics SAGE IBM® SPSS® Statistics 23 software was used to analyze the data (supplied by SPSS Inc., USA) and findings are presented as mean ± SD. The distribution of variables was normal as assessed by Kurtosis-Skewness statistics. Independent t test was used for comparing primary evaluations of all variables in the two groups at the baseline and (χ2) criterion was also used for categorical and numeric variables. Changes in body measurements and blood parameters of patients were measured between pre-test and posttest by paired-samples t test. Analysis of covariance (ANCOVA) was applied to recognize any discrepancies between the two groups after the supplementation, adjusting for baseline measurements and confounders. Repeated measures ANOVA were exerted to assess the within-group changes in dietary intake. The following equation determined the variable alterations after intervention by percentage: [(subtraction of after and before values) divided by before values] multiplied by 100. Statistical significance was considered at P<0.05.

## Results

**Fig.1:**
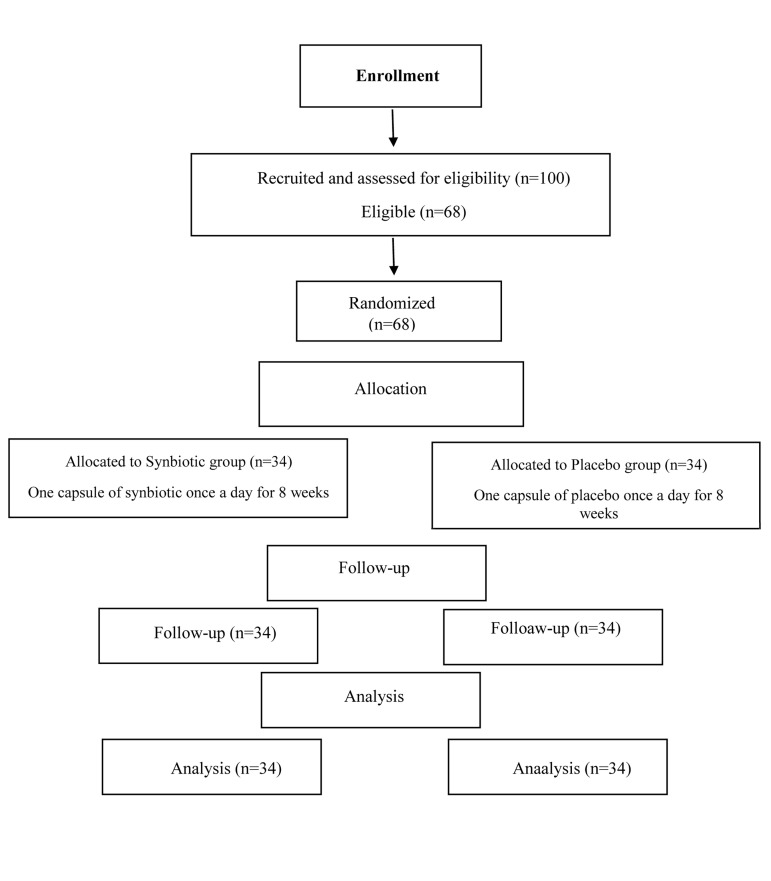
Participants flow diagram.

All participants [the synbiotic group (n=34) as well as the placebo group (n=34)] were ended the study (Fig .1). The adoption of the study was performed well and 95% of the prescribed supplements were consumed during the study. No complication or symptoms were reported following supplementation.

Public characteristics of the patients are shown in Table 1. There were no significant discrepancies between the two groups in the means of age, weight, BMI and physical activity levels at baseline. No groups had significant changes (P<0.05) in the rate of women’s physical activity during the study.

**Table 1:** General features of women with PCOS participated in this trial


Variable	Measurement period	Placebo groupn=34	Synbiotic groupn=34	MD (95% CI), P value

Age (Y)	Baseline	28.6 ± 4.82	30.4 ± 5.82	
Weight (kg)	Baseline	73.67 ± 10.89	76.15 ± 14.97	2.47 (-3.8 to 8.82), 0.438
BMI (kg/m^2^)	Baseline	28.47 ± 3.55	29.43 ± 5.69	0.95 (-1.34 to 3.26), 0.409
Physical activity	Baseline			0.564^*^
	Low	19 (55.9)	16 (47.1)	
	Moderate	8 (23.5)	14 (41.2)	
	High	7 (20.6)	4 (11.8)	
	After intervention			0.328^*^
	Low	18 (52.9)	14 (41.2)	
	Moderate	9 (26.6)	15 (44.1)	
	High	7 (20.5)	5 (14.7)	


Data are presented as mean ± SD or n (%). PCOS; Polycystic ovary syndrome, CI; Confidence interval, MD; Means difference, BMI; Body mass index, and *; P value is reported based on the chi-square test. MD (95% CI); P value is reported based on the analysis of independent sample t test.

**Table 2:** Dietary intakes of women with PCOS participated in this trial at baseline and after 8 weeks of intervention


Variable	Measurement period	Placebo group n=34	Synbiotic groupn=34	P value

Energy (kcal/day)	Before	1528.19 ± 300.87	1759.08 ± 511.30	0.027^*^
	Fourth week	1682.55 ± 360.35	1847.06 ± 387.35	0.517^*^^*^
	After	1636.36 ± 286.46	1796.08 ± 423.39	0.566^*^^*^
	P value^†^	0.021	0.391	
Carbohydrate (g/day)	Before	217.46 ± 50.16	257.47 ± 94.69	0.033^*^
	Fourth week	230.94 ± 60.64	259.65 ± 59.78	0.347
	After	235.58 ± 54.12	256 ± 67.51	0.804
	P value	0.143	0.954	
Protein (g/day)	Before	51.40 ± 12.74	58.54 ± 20.83	0.093
	Fourth week	56.68 ± 15.68	61.78 ± 14.89	0.610
	After	53.06 ± 13.70	60.17 ± 15.46	0.207
	P value	0.109	0.519	
Total fat (g/day)	Before	52.80 ± 14.69	57.31 ± 15.48	0.223
	Fourth week	61.68 ± 16.83	64.18 ± 22.86	0.905
	After	56.04 ± 12.84	62.03 ± 20.70	0.316
	P value	0.015	0.097	


Data are presented as mean ± SD. PCOS; Polycystic ovary syndrome, *; Significant difference between the two groups at baseline (P<0.05, independent sample t test), † ; P value is reported based on the analysis of the repeated measures, and **; P value is reported based on analysis of covariance (adjusted for baseline values).

**Table 3:** Biochemical parameters of women with PCOS participated in this trial, at baseline and after 8 weeks intervention


Variable	Measurement period	Placebo group n=34	Synbiotic group n=34	MD (95% CI), P value

Glucose(mg/dL)	Baseline	89.02 ± 9.05	91.32 ± 8.07	2.29 (-1.86 to 6.45), 0.274
	After intervention	94.44 ± 9.49	90.08 ± 7.90	-4.52 (-8.50 to -0.54), 0.026^†^
	MD (95% CI), P value	5.41 (2.38 to 8.43), 0.001^*^^*^	-1.2 (-4.58 to 2.11), 0.459	
Insulin (μIU/mL)	Baseline	9.46 ± 4.64	13.36 ± 4.89	3.89 (1.58 to 6.20), 0.001^*^
	After intervention	13.17 ± 5.29	11.50 ± 4.75	-3.90 (-6.18 to -1.62), 0.001^†^
	MD (95% CI), P value	3.70 (2.06 to 5.34), 0.000^*^^*^	-1.85 (-3.41 to -0.29), 0.021^*^^*^	
HOMA-IR	Baseline	2.10 ± 1.12	3.06 ± 1.35	0.95 (0.35 to 1.55), 0.002^*^
	After intervention	3.08 ± 1.31	2.58 ± 1.15	-0.93 (-1.50 to -0.36),0.001^†^
	MD (95% CI), P value	0.97 (0.56 to 1.39), 0.000^*^^*^	-0.47 (-0.90 to -0.04), 0.032^*^^*^	
TC (mg/dL)	Baseline	197.91 ± 39.80	209.41 ± 33.16	11.50 (-6.25 to 29.25), 0.200
	After intervention	210.11 ± 39.17	208.55 ± 38.92	-6.93 (-22.58 to 8.71), 0.379
	MD (95% CI), P value	12.20 (3.09 to 21.31), 0.010^*^^*^	-0.85 (-13.92 to 12.21), 0.895	
TG (mg/dL)	Baseline	140.76 ± 71.22	139.29 ± 69.92	-1.47 (-35.64 to 32.70), 0.932
	After intervention	149.14 ± 83.83	137.97 ± 68.61	-11.88 (-31.79 to 8.03), 0.238
	MD (95% CI), P value	8.38 (-5.23 to 22), 0.219	-1.32 (-15.20 to 12.55), 0.847	
LDL-cholesterol (mg/dL)	Baseline	121.61 ± 31.64	135.75 ± 28.40	14.14 (-0.41 to 28.71), 0.057
	After intervention	136.05 ± 32.60	133.84 ± 36.07	-8.34 (-24.11 to 7.42), 0.294
	MD (95% CI), P value	14.44 (5.77 to 23.10), 0.002^*^^*^	-1.91 (-14.74 to 10.92), 0.764	
HDL-cholesterol (mg/dL)	Baseline	48.14 ± 10.22	45.79 ± 12.05	-2.35 (-7.76 to 3.06), 0.389
	After intervention	44.23 ± 10.73	47.11 ± 12.73	5.39 (0.74 to 10.05), 0.024^†^
	MD (95% CI), P value	-3.91 (-7.91 to 0.09), 0.055	1.32 (-1.25 to 3.90), 0.304	
Apelin (nmol/mL)	Baseline	28.12 ± 23.56	20.06 ± 13.78	-8.06 (-19.53 to 3.41), 0.164
	After intervention	32.93 ± 25.88	21.86 ± 14.87	-0.81 (-10.88 to 9.25), 0.871
	MD (95% CI), P value	4.80 (-0.96 to10.57), 0.098	1.80 (-5.47 to 9.07), 0.613	


Data are presented as mean ± SD. PCOS; Polycystic ovary syndrome, CI; Confidence interval, MD; Means difference, HOMA-IR; Homeostatic model assessment for insulin resistance, TC; Total cholesterol, TG; Triglyceride, LDL; Low-density lipoprotein, HDL; High-density lipoprotein, BMI; Body mass index, *; P value is reported based on the analysis of independent sample t test, **; P value is reported based on the analysis of paired sample t test, and † ; P value is reported based on the analysis of covariance, adjusted for energy intake, BMI and baseline values.

Daily dietary intakes of patients throughout the study are shown in Table 2. There were significant differences (P<0.05) between the two groups in average of daily energy and carbohydrate intakes at baseline. Diversities at other macronutrients intake were not notable between the two groups at baseline. There was a significant increase in energy and whole lipid intake in the placebo group during the research (P=0.02 and P=0.01, respectively). Changes in dietary intakes were not considerable in the synbiotic group. No significant differences were detected in energy and macronutrients intakes between the two groups at the end of the trial (P>0.05).

Metabolic parameters of patients at the beginning and after 8-weeks supplementation are shown in Table 3. There were significant distinctions between the two groups in means of serum insulin (P=0.001) and HOMAIR (P=0.002) at baseline. No significant differences were seen between the two groups in levels of other biomarkers at baseline.

Results of analysis of covariance indicated statistically considerable variations between the two studied groups in serum levels of glucose (P=0.02), insulin (P=0.001), HOMA-IR (P=0.001) and HDL cholesterol (P=0.02) finally, set toward energy intake, BMI as well as initial amounts. There were no significant alterations in blood TC, TG, LDL cholesterol and apelin levels. 

Supplementation with symbiotic reduced by respectively 1.35, 13.92 and 15.68% at blood ratios of glucose, insulin and HOMA-IR and 2.88% increase in HDL cholesterol, in comparison to the placebo group.

Table 3 shows a substantial reduction in insulin and HOMA-IR (by 13.92%, P=0.02 and 15.68%, P=0.03, respectively) in the synbiotic group at the end of the trial in comparison to the baseline values. Also, serum ratios for glucose increased within the placebo group (by 6.08 %, P=0.001). Serum apelin concentrations stayed unchanged in the two groups at the end of the study. The baseline and post-intervention values for apelin levels which had a wide SD were 28.12 ± 23.56 nmol/mL and 32.93 ± 25.88 nmol/ mL in the placebo group and 20.06 ± 13.78 nmol/mL and 21.86 ± 14.87 nmol/mL in the synbiotic group, respectively.

Anthropometric measurements of women with PCOS at baseline and after 8-weeks supplementation are shown in Table 4. There were no considerable differences between the two groups in weight, BMI, WC, HC, WHR and WHtR at baseline.

**Table 4:** Anthropometric characteristics of subjects with PCOS participated in this trial, at baseline and after 8 weeks of intervention


Variable	Measurement period	Placebo groupn=34	Synbiotic groupn=34	MD (95% CI), P value

Weight (kg)	Baseline	73.67 ± 10.89	76.15 ± 14.97	2.47 (-3.8 to 8.82), 0.438
	After intervention	74.22 ± 11.14	75.08 ± 15.35	-1.58 (-2.91 to -0.24),0.021^†^
	MD (95% CI), P value	0.55 (0.19 to 0.90), 0.003^*^^*^	-1.07 (-2.36 to 0.21), 0.099	
BMI (kg/m^2^)	Baseline	28.47 ± 3.55	29.43 ± 5.69	0.95 (-1.34 to 3.26), 0.409
	After intervention	28.72 ± 3.63	29.00 ± 5.76	-0.63 (-1.18 to -0.09), 0.021^†^
	MD (95% CI), P value	0.24 (0.08 to 0.39), 0.003^*^^*^	-0.43 (-0.94 to 0.08), 0.101	
WC (cm)	Baseline	93.08 ± 11.49	93.44 ± 11.77	0.35 (-5.28 to 5.98) , 0.901
	After intervention	93.75 ± 11.71	91.08 ± 11.41	-2.94 (-5.25 to -0.64), 0.013^†^
	MD (95% CI), P value	0.66 (-0.93 to 2.25), 0.406	-2.35 (-4.09 to -0.61), 0.009^*^^*^	
HC (cm)	Baseline	108.79 ± 7.74	110.19 ± 10.69	1.39 (-3.13 to 5.92), 0.540
	After intervention	109.32 ± 8.27	108.86 ± 11.01	-1.81 (-3.38 to -0.24), 0.024^†^
	MD (95% CI), P value	0.52 (-0.24 to 1.30), 0.176	-1.32 (-2.68 to 0.03), 0.056	
WHR	Baseline	0.85 ± 0.06	0.84 ± 0.05	-0.007 (-0.03 to 0.02), 0.643
	After intervention	0.85 ± 0.07	0.83 ± 0.05	-0.01 (-0.03 to 0.00), 0.110
	MD (95% CI), P value	0.00 (-0.01 to 0.01), 0.678	-0.01 (-0.02 to 0.00), 0.090	
WHtR	Baseline	0.57 ± 0.06	0.57 ± 0.07	0.00 (-0.03 to 0.03), 0.974
	After intervention	0.57 ± 0.07	0.56 ± 0.07	-0.01 (-0.03 to -0.00), 0.027^†^
	MD (95% CI), P value	0.00 (-0.00 to 0.01), 0.460	-0.01 (-0.02 to -0.00), 0.028^*^^*^	


Data are presented as mean ± SD. PCOS; Polycystic ovary syndrome, CI; Confidence interval, MD; Means difference, BMI; Body mass index, WC; Waist circumference, HC; Hip circumference, WHR; Waist to hip ratio, WHtR; Waist- to- height ratio, **; P value is reported based on the analysis of, paired sample t test, and † ; P value is reported based on the analysis of covariance, adjusted for energy intake and baseline values.

Results of analysis of covariance illustrated a statistically significant discrepancy between the two studied groups in weight (P=0.02), BMI (P=0.02), WC and HC (P=0.01, P=0.02, respectively) and WHtR (P=0.02) at the end of the study, adjusted for energy intake and baseline values. Changes in WHR was not significant between the two groups at the end of the study (P>0.05).

Significant decreases (by 2.52% and 1.75%, respectively) were observed in WC and WHtR of subjects in the synbiotic group after the intervention compared to the baseline values (P=0.009 and P=0.02, respectively). Changes in other anthropometric variables were not significant within the synbiotic group. Weight and BMI increased within the placebo group (respectively by 0.74%, P=0.003 and 0.87%, P=0.003).

## Discussion

The application of probiotics and prebiotics can ameliorate the contrast between intestinal microbiota and host metabolism in obesity and associated metabolic diseases (28). Few studies assessed potential influences of synbiotics in subjects with PCOS. To the best of our knowledge, impacts of synbiotics on lipids profile and obesity values have not been investigated in subjects with PCOS by a supplement similar to that used in our study with respect to form, dose, strains, and duration of use. In a previous study about synbiotic supplementation in PCOS patients that assessed glycemic indices and apelin levels,1000 mg dosage of the capsule (20) was used, which was different from our study (i.e. 500 mg).

According to findings of present trial, synbiotic supplementation reduced fasting blood glucose, HOMA-IR and insulin in patients during eight weeks of supplementation. Our results are in accordance with the findings reported by Samimi et al. (19) which showed that the use of one synbioticcapsules (genera *Lactobacillus Lactobacillus acidophilus, L. casei,* and *B. bifidum,* 2×10^9^ CFU/g together with 800 mg inulin) per day for 12 weeks, reduced serum insulin and HOMA-IR in PCOS women. Esmaeilinezhad et al. (29) reported that consumption of synbiotic pomegranate juice and synbiotic beverage (2 L/week) for 8 weeks, lowered HOMA-IR in women with PCOS.

It was suggested that synbiotics may play a momentous role in the metabolism of carbohydrates by producing short-chain fatty acids (SCFAs). SCFAs bind to G protein-coupled receptors and increase the secretion of glucagon-like peptide 1 (GLP-1) and peptide YY (PYY), from enteroendocrine L-cells which can trigger insulin production by pancreatic β cells, inhibit glucagon secretion, decrease hepatic gluconeogenesis, and raise insulin sensitivity (17). Synbiotics also improve bowel function, elevate the production of mucin and diminish the amount of gram-negative (inappropriate) bacteria in the colon. These changes decrease the transmission of lipopolysaccharides (LPS) along the mucous wall and metabolic endo-toxaemia, which can ultimately lead to improvements in insulin receptor function, lower insulin levels, and increased normal ovarian function (15).

Our results confirmed improving effects of synbiotic administration on glycemic indices by lowering HOMAIR and subsequent lowered blood glucose and insulin in the studied women. However, in a study by Karimi et al. (20) use of synbiotic supplement did not affect these parameters in women with PCOS. Non-effectiveness of synbiotics on glycemic indices has been also reported in a study on subjects with nonalcoholic steatohepatitis (30). Differences in clinical and metabolic characteristics of participants, as well as varying strains and doses of probiotics and type of prebiotics, treatment period and host gut microbiota, might contribute to controversial findings.

HDL cholesterol is considered the helpful cholesterol due to its function of transporting cholesterol in the shape of cholesteryl esters to the liver for rather a hydrolysis (31). According to our results, the mean serum HDL cholesterol levels of studied subjects were lower than 50 mg/dl (normal cut-off based on National Cholesterol Education Program Adult Treatment Panel III) (32) in both groups at baseline and its level elevated in the synbiotic group at the end of the study in comparison with the placebo group. Samimi et al. (19) in their research concluded that the intervention with symbiotic for 12 weeks in PCOS patients, had significant alleviations of serum TG and VLDL cholesterol levels. No other study is available about possible effects of synbiotics on lipid profiles in PCOS.

It was proposed that probiotics may interfere in the removal of cholesterol by reducing cholesterol absorption from the intestine (33). Liong and Shah (34) showed that cholesterol conjugation to the cell wall of probiotics and their special abilities in enzymatic biliary acid decontamination lead to a decrement of serum cholesterol. Moreover, it was demonstrated that the cholesterollowering effects of probiotics increase with the use of prebiotics, concurrently (33).

In the present study, along with improvement in serum HDL cholesterol in the synbiotic group, no significant change was detected in other serum lipids. So, further studies are needed to investigate longer synbiotics administration effects and their precise effects on lipid metabolism. 

Obesity displays a considerable role in the progress of metabolic disease in women with PCOS. Studies have indicated that the gut microbiota as an environmental factor was altered in obesity and leads to its spread (35, 36).

Our results indicated that synbiotic supplementation reduced weight and BMI and central obesity indices including WC, HC, and WHtR in the intervened group compared to the placebo group. As previously mentioned, no other study investigated possible effects of synbiotics in the form of our supplement on anthropometric characteristics in women with PCOS.

Studies showed that intestinal microbiota modifies the biological system, which results in the regulation of nutrient availability, energy storage, spread of fat mass and inflammation in the host, both of which are associated with obesity (17). The intestinal microbiota is also effective in regulating nutrient intake via the SCFA signaling function (36).

Women participated in our research were asked to follow their previous diet and physical activity, and our analysis showed that there were no significant changes in these variables during the intervention. As a result, it seems that improved obesity values in the group intervened with synbiotics, were not induced by changes in food intake or physical activity. It was possible that the reduction in obesity values in the synbiotic group might be related to improvement in HOMA-IR, at least in part. Evidence suggests that higher insulin sensitivity reduces hyperglycemia, hepatic lipid synthesis and fat accumulation in adipose tissues (37). Additionally, changes in anthropometric measurements were not mediated through apelin levels. Since, synbiotic supplementation did not affect serum apelin levels in our study

It was suggested that synbiotics by changing the balance of intestinal microbiota (DOGMA), may affect endocannabinoid and apelinergic system. Intestinal microbiota imbalance enhances the permeability of the intestinal epithelium and consequently, the influx of LPS into the circulation, which finally leads to metabolic endotoxemia, activation of the immune system and induction of inflammation. These conditions promote the activity of the apelinergic system in the adipose tissue and the level of apelin in the serum. Thus, changes in intestinal microbiota with symbiotic play an important role in reducing apelin levels in PCOS patients (38). Karimi et al. (20) reported a significant decrease in serum apelin levels in women with PCOS following synbiotic supplementation from 27 ± 21 nmol/l at baseline to 14.4 ± 4.5 nmol/l at the end of study. Changes in the placebo group were not significant (26 ±15 nmol/l and 18.4 ± 2.9 nmol/l, at the beginning and the end of study, respectively). In present study, as described in results section, wide SD distribution of apelin might have contributed to nonconsiderable changes in concentration of this adipokine. It was also probable that dose or duration of supplementation was not adequate to affect the aplin levels in our trial. As mentioned previously, Karimi et al. (20) applied a twotime higher supplement dosage (1000 mg) for a longer period (12 weeks) and obtained significant changes in apelin levels. No other study is available about possible effects of synbiotics on apelin levels in PCOS or other diseases.

It should be noted that the exact normal range for serum apelin has not been documented yet. To date, a few studies have measured apelin in subjects with PCOS, and their results are incompatible. In the study by OlszaneckaGlinianowic et al. (24) no significant difference was seen in serum levels of this adipokine in PCOS and non-PCOS women. Plasma apelin-36 levels was significantly higher in normal-weight women than the obese PCOS women (3.1 ± 2.2 vs. 1.2 ± 0.7 μg/l, respectively). In another study, serum apelin levels were correlated positively with BMI, IR, serum insulin and TG in women with PCOS. Apelin levels were lower in women with PCOS than controls (194.1 ± 50.7 pg/ml vs. 292.1 ± 85.6 pg/ml, respectively) (39). Inconsistent results among the findings of the available studies may be related to the design and the demographic and genetic specifications of populations as well as the nature of apelin. 

In our study, no association was found between serum apelin concentrations and values of obesity or biochemical parameters before or after interventions in either group (data are not shown). As a result, it seems that favorable changes detected in glycemic indices and lipids profile in our study were not mediated via apelin. Subsequent studies are needed to evaluate the intestinal microbiota impacts on circulating apelin as well as the role of this adipokine in the pathogenesis of PCOS.

The strength of our study was the double-blind placebocontrolled design with no drop-outs. However, the present study had some limitations such as its short study duration of 8 weeks. Also, bacterial flora changes and SCFAs were not assessed through analysis of the stool. This research included overweight or obese patients. Therefore, our findings cannot be generalized to low-weight and/ or normal-weight PCOS women, various intervention periods and other kinds of synbiotics. Additional studies are warranted to identify the impacts of synbiotics on other serum adipokines and androgen status in women with PCOS.

## Conclusion

It can be said that synbiotic supplementation improved glycemic indices, serum HDL cholesterol levels and obesity values in subjects with PCOS and may be useful in the control of metabolic factors and reducing adiposity in these patients. Synbiotic administration in this study did not affect serum apelin levels. It is offered that the physiopathological function of apelin and metabolic effects of synbiotics in PCOS patients be evaluated more in future studies.
